# CO_2_ Laser versus Sham Control for the Management of Genitourinary Syndrome of Menopause: A Systematic Review and Meta-Analysis of Randomized Controlled Trials

**DOI:** 10.3390/jpm13121694

**Published:** 2023-12-08

**Authors:** Anastasia Prodromidou, Dimitrios Zacharakis, Stavros Athanasiou, Nikolaos Kathopoulis, Antonia Varthaliti, Athanasios Douligeris, Lina Michala, Veatriki Athanasiou, Stefano Salvatore, Themos Grigoriadis

**Affiliations:** 1 1st Department of Obstetrics and Gynaecology, Alexandra Hospital, National and Kapodistrian University of Athens, 11528 Athens, Greece; dimzac@hotmail.com (D.Z.); athanasio@med.uoa.gr (S.A.); nickatho@gmail.com (N.K.); antonia.varthaliti@hotmail.com (A.V.); thanosdouligeris92@gmail.com (A.D.); linamichala@med.uoa.gr (L.M.); tgregos@med.uoa.gr (T.G.); 2 Medicine, Brighton and Sussex Medical School, Brighton BN1 9PX, UK; beatrice.ath@gmail.com; 3 Obstetrics and Gynaecology Department, IRRCS San Raffaele Hospital, Vita-Salute San Raffaele University, 20125 Milan, Italy; salavatore.stefano@hsr.it

**Keywords:** CO_2_ vaginal laser, genitourinary syndrome of menopause, vaginal atrophy, dyspareunia

## Abstract

In the context of the menopausal transition, genitourinary syndrome of menopause (GSM) refers to a range of genitourinary symptoms, from vaginal dryness to dysuria and urinary urgency. While hormonal treatments are standard, their associated side effects have driven the exploration of alternatives like vaginal CO_2_ laser. We aimed to evaluate the randomized controlled trials (RCTs) comparing vaginal CO_2_ laser treatment for GSM to sham controls. This systematic review sourced four electronic databases until June 2023. The analysis incorporated seven RCTs with 407 women. The CO_2_ laser and sham control were comparable for most parameters, including the female sexual function index (FSFI) and visual analogue scale (VAS) for dyspareunia, vaginal health index, pH, and patient satisfaction. However, the CO_2_ laser group showed significant improvement in the vaginal assessment scale for GSM symptoms. Sensitivity analyses revealed that parameters like FSFI showed significant differences in favor of CO_2_ laser group upon the exclusion of specific studies. In conclusion, vaginal CO_2_ laser therapy emerges as a promising alternative for GSM management, especially for most bothersome GSM symptoms; however, the need for further well-designed RCTs remains to validate its broad safety and efficacy.

## 1. Introduction

In 2014, the International Society for the Study of Women’s Sexual Health (ISSWSH) and the North American Menopause Society (NAMS) introduced the term genitourinary syndrome of menopause (GSM) to describe the genitourinary symptoms that are related to the menopausal transition [1]. Among the clinical manifestations of GSM, the most common ones include vaginal dryness, irritation, reduced lubrication, dyspareunia, decreased libido, dysuria, urinary frequency/urgency, and recurrent urinary tract infections. GSM was previously referred to as vulvovaginal atrophy or atrophic vaginitis, but the term GSM is preferred as it comprehensively encompasses the wide range of symptoms and signs related to estrogen deficiency in the genitourinary tract [1]. In the literature, the reported prevalence of GSM has been found to range between 50% and 70% [2].

First-line treatment options of GSM include various non-hormonal therapies, such as vaginal lubricants and moisturizers. Should these fail, the second-line treatment options include hormonal treatment in the form of local estrogens aiming to replenish the lost estrogen [3]. However, estrogen-based treatments have been related to side effects including breast tenderness, vaginal bleeding, bloating, and mood fluctuations while they are contraindicated in women with a history of gynecological cancer, coronary artery disease, venous thromboembolism, stroke, liver disease, and unexplained vaginal bleeding. Due to these concerns, researchers have explored alternative treatments [4]. In this context, vaginal CO_2_ laser has been proposed as a potential therapeutic modality for the management of GSM. Its effectiveness is attributed to the process of collagen denaturation and induced neocollagenesis, aiming to improve the integrity of the vaginal epithelium, subepithelial fascia, and connective tissue [5]. The currently available literature includes a plethora of studies, respective reviews, and meta-analyses evaluating the efficacy of laser CO_2_. However, the majority of them represent observational single-arm studies and studies (either RCTs or observational) comparing laser CO_2_ with other therapeutic modalities, including vaginal hormonal therapy or Kegel exercises [6,7]. Therefore, the evaluation of the potential placebo effect is still limited to a small number of RCTs comparing laser CO_2_ with sham control and to one meta-analysis evaluating half of the currently available RCTs [8].

The aim of the present systematic review and meta-analysis is to examine the efficacy of the use of a vaginal CO_2_ laser in the management of GSM compared with sham control (placebo) based on the outcomes from randomized controlled trial (RCT) studies.

## 2. Materials and Methods

### 2.1. Study Design and Eligibility Criteria

The updated guidelines for Systematic Reviews and Meta-analyses (PRISMA) were used for the design of the present meta-analysis according to the authors’ predetermined inclusion criteria [9]. No language restrictions were applied. All randomized controlled trials (RCTs) that evaluated the effect of vaginal CO_2_ laser in patients with GSM compared with a sham control were assessed and critically appraised. Only comparative RCTs that reported at least one of the predetermined primary outcomes were considered eligible. Non-randomized trials, letters to the editor, editorials, conference papers, case reports, reviews, and animal experimental studies were excluded from tabulation and analysis. Patient consent and the Institutional review board were not applicable for this type of study. The study’s protocol was published in Open Science Framework (doi:10.17605/osf.io/5d8qy).

The inclusion criteria were as follows: female patient aged >18 years; menopausal status defined as amenorrhea > 12 months (iatrogenic or natural); at least one vaginal symptom including dryness, burning, dyspareunia, and/or itching. Studies that included patients who received other treatment modalities such as estrogen therapy, or other energy-based devices, and were compared to those who had laser CO_2_ were excluded. Additionally, the comparison of outcomes before and after laser treatment was also considered a criterion for exclusion.

### 2.2. Information Sources

The literature search was systematic and performed in three stages. Initially, four electronic databases—Medline (1966–2023), Scopus (2004–2023), Cochrane CENTRAL Register of Controlled Trials, and Clinicaltrials.gov—were searched until June 2023. The date of the last search was 27 November 2023. Titles and/or abstracts of comparative studies that evaluated the outcomes of patients who received CO_2_ laser intravaginal therapy were assessed for eligibility. Studies that were deemed to meet criteria were retrieved in full text. Additionally, the references of the eligible articles were also searched for further relevant studies in the field. A minimum number of keywords was utilized in an attempt to assess a number of studies that could be easily searched while simultaneously minimizing the potential loss of articles. The following keywords were utilized: “laser CO_2_”, “genitourinary syndrome of menopause”, “fractional laser”, “vulvovaginal atrophy”, and “sham control”.

### 2.3. Search

The search was performed using the keywords and Boolean operators. Our search strategy in PubMed used the following search terms:

The PICO criteria that were used to develop our search strategy were as follows:

Patient/ Problem: Female adult menopausal patients with GSM, Intervention: vaginal laser CO_2_, Comparison: vaginal laser CO_2_ versus sham control, Outcome: female sexual function index (FSFI), dyspareunia according to visual analogue scale (VAS) score, GSM symptoms according to vaginal assessment scale.

- “lasers, gas”[MeSH Terms] OR (“lasers”[All Fields] AND “gas”[All Fields]) OR “gas lasers”[All Fields] OR (“laser”[All Fields] AND “co2”[All Fields]) OR “laser co2”[All Fields]) AND (“vagina”[MeSH Terms] OR “vagina”[All Fields] OR “vaginal”[All Fields] OR “vaginally”[All Fields] OR “vaginals”[All Fields] OR “vaginitis”[MeSH Terms] OR “vaginitis”[All Fields] OR “vaginitides”[All Fields]) AND (“atrophie”[All Fields] OR “atrophy”[MeSH Terms] OR “atrophy”[All Fields] OR “atrophied”[All Fields] OR “atrophies”[All Fields] OR “atrophying”[All Fields].
- “urogenital system”[MeSH Terms] OR (“urogenital”[All Fields] AND “system”[All Fields]) OR “urogenital system”[All Fields] OR “genitourinary”[All Fields]) AND (“syndrom”[All Fields] OR “syndromal”[All Fields] OR “syndromally”[All Fields] OR “syndrome”[MeSH Terms] OR “syndrome”[All Fields] OR “syndromes”[All Fields] OR “syndrome s”[All Fields] OR “syndromic”[All Fields] OR “syndroms”[All Fields]) AND (“menopause”[MeSH Terms] OR “menopause”[All Fields] OR “menopausal”[All Fields] OR “menopaused”[All Fields] OR “menopauses”[All Fields]) AND (“laser s”[All Fields] OR “lasers”[MeSH Terms] OR “lasers”[All Fields] OR “laser”[All Fields] OR “lasered”[All Fields] OR “lasering”[All Fields].

### 2.4. Selection of Sources of Evidence

**Figure 1:**
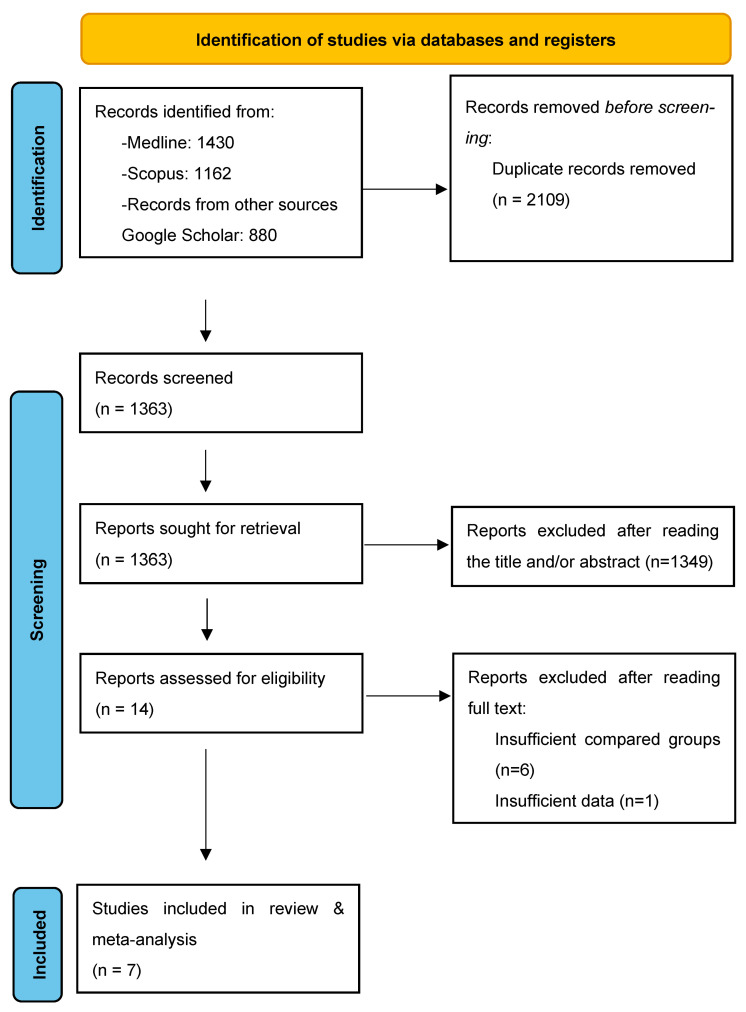
Search flow diagram.

The studies were initially selected based on their title and then on their abstract in case of eligibility ambiguities. After exclusion of duplicates, the predetermined inclusion and exclusion criteria were applied. Articles that fulfilled or were deemed to fulfil the inclusion criteria were retrieved. Three authors (DZ, TG, and AP) performed an independent and meticulous search of the literature, excluded overlaps, and tabulated the selected indices in structured forms. The discrepancy among the authors was discussed by all of them until they reached a consensus. The PRISMA flow diagram schematically presents the stages of article selection (Figure 1).

#### Data-Charting Process, Data Items, and Synthesis of Results

All authors discussed the variables to be extracted by the included studies, and structured tables were independently fulfilled by two of them (AP and DZ). After extraction, the validity of the extracted data was discussed and potential discrepancies were dissolved in order to achieve accuracy and validity. Data on patient characteristics included patients’ age, parity, sexual activity, years in menopause and use of lubrication and/or hormone replacement therapy (HRT). Our primary outcomes were as follows: visual analogue scale (VAS) for dyspareunia and vaginal assessment scale for GSM symptoms, female sexual function index (FSFI), vaginal health index (VHI), patient satisfaction, and vaginal pH. Among them, in case of the VAS, vaginal assessment scale, FSFI, VHI, and vaginal pH, we assessed the difference between the outcomes at baseline and follow-up, while for the assessment of patient satisfaction we sought to evaluate the proportion of participants who reported being “satisfied” or “very satisfied” with the procedure. Finally, vaginal assessment scale for GSM symptoms is a tool that was used to assess vaginal symptoms including dryness, soreness, irritation, and dyspareunia, with scores ranging from 0 (none) to 3 (severe) and the final score being derived from the average of scores for each domain.

### 2.5. Quality Assessment

**Figure 2:**
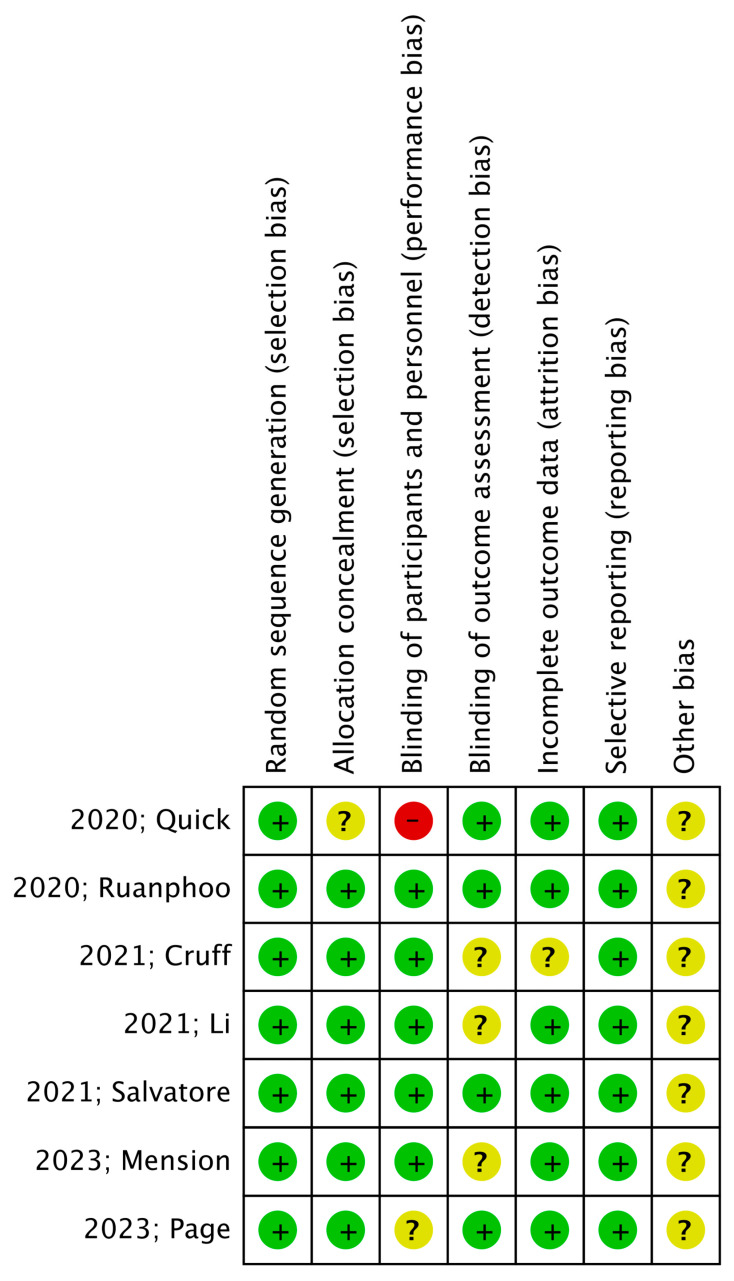
RCT’s methodological quality. Risk-of-bias summary: “−” represents high risk of bias, “?” represents unclear bias, and “+” represents low risk of bias [12,13,14,15,16,17,18].

For the evaluation of the methodological quality of the recruited studies, the Review Manager 5.4 tool for the assessment of the “Risk of Bias” was utilized, according to the Cochrane Collaboration Handbook (Figure 2) [10]. Two authors (DZ and AP) independently performed the procedure. The Grading for Recommendations Assessment, Development, and Evaluation (GRADE) framework was used for assessment of the quality of evidence for primary outcomes that could range from very low to high [11]. In particular, credibility of evidence was assessed by taking into consideration the following domains: study limitations, directness, consistency, precision, and publication bias.

### 2.6. Statistical Analysis

Statistical meta-analysis was performed using the RevMan 5.4 software (Copenhagen: The Nordic Cochrane Centre, The Cochrane Collaboration, 2011). Confidence intervals (CI) were set at 95%. Mean difference (MD) and odds ratios (OR) were used in the analysis. The results were calculated using the DerSimonian–Laird random effect model (REM), revealing significant heterogeneity in the methodological characteristics of the included studies [19]. Publication bias was not tested due to the significant heterogeneity of the included studies, which may influence the methodological integrity of the tests and their limited number (n < 10 studies). Mean values and standard deviations were calculated according to the equations proposed by Hozo et al. when not provided by the studies [20]. The cut-off for statistical significance was set at *p* < 0.05.

### 2.7. Sensitivity Analysis

A sensitivity analysis was performed by excluding one study at a time with the intention of examining whether outcomes of selected studies could result in an alteration in the outcomes of the meta-analysis.

## 3. Results

### 3.1. Excluded Studies

A total of seven studies were excluded from tabulation and analysis [21,22,23,24,25,26,27]. More specifically, the study by Lou et al. compared patients who were randomized with those who received laser and those who performed Kegel exercise training [21]. The study by Li et al. was excluded due to insufficient data [22], while the remaining five studies did not include patients who had sham treatment as control and used patients who received topical estrogen or lubricants for comparison with the laser CO_2_ group [23,24,25,26,27].

### 3.2. Included Studies

A total of seven RCTs that recorded outcomes of 407 women suffering from GSM were finally included in the present meta-analysis [12,13,14,15,16,17,18]. Among them, 201 received treatment with vaginal CO_2_ laser, while the remaining 206 were enrolled in the sham control group that received the same procedure with the laser without the activation of the device. The analyzed indices were tabulated in two structured tables as follows: Table 1 depicts the main characteristics of the included studies and patients, and Table 2 shows the main outcomes from the included studies.

### 3.3. Quality Assessment

The assessment of the methodological quality of the included studies according to the “Risk of Bias” assessment tool is depicted in Figure 2. In particular, the selection bias was low as all of the studies were randomized and double-blinded, thus reducing the performance bias. Detection and attrition bias were low, while reporting bias was moderate. Therefore, the aforementioned parameters provide a high general study quality. GRADE assessment for the evaluation of evidence for primary outcomes revealed that regarding VAS scores for dyspareunia, FSFI, VHI, and vaginal pH, the overall quality was moderate. Finally, regarding the vaginal assessment scale for GSM symptoms and patient satisfaction, the overall quality was evaluated as high.

### 3.4. Patient Characteristics

Mean patients’ age ranged from 51.3 to 61.7 and from 53.7 to 59.8 in the laser and control groups, respectively. Follow-ups ranged from 3 to 12 months.

### 3.5. Main Outcomes

**Figure 3:**
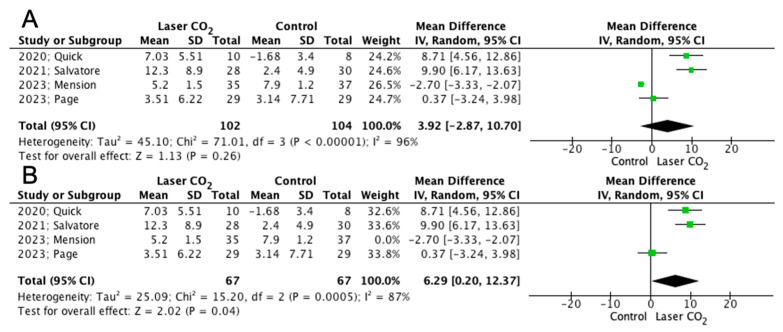
Forest plot depicting mean difference in FSFI in (**A**) overall and (**B**) sensitivity analysis [12,16,17,18].

Table 3 depicts the main outcomes reported from the included studies. Despite the fact that mean differences in FSFI scores were not different among the two groups, sensitivity analysis revealed a significantly increased mean FSFI difference after treatment in favor of laser CO_2_ when the study by Mension et al. was excluded from analysis (206 patients: MD 3.92; 95% CI −2.87, 10.70; *p* = 0.26; 134 patients: MD 6.29; 95% CI 0.20, 12.37; *p* = 0.04, respectively), as shown in Figure 3.

**Figure 4:**
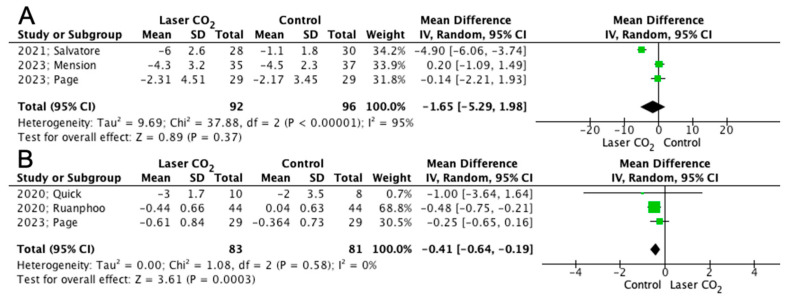
Forest plot depicting mean difference in (**A**) VAS for dyspareunia and (**B**) vaginal assessment scale for GSM symptoms [12,13,16,17,18].

The mean difference in VAS scores for dyspareunia after treatment was comparable between patients who received laser treatment and those from the sham control group (188 patients: MD −1.65; 95% CI −5.29, 1.98; *p* = 0.37; Figure 4A). However, the mean difference in vaginal assessment scale concerning the improvement of GSM symptoms was significantly higher in favor of the laser CO_2_ group (164 patients: MD −0.41; 95% CI −0.64, −0.19; *p* = 0.0003; Figure 4B).

Neither VHI nor vaginal pH were found to be different among the two groups (218 patients: MD −0.83; 95% CI −2.76, 1.10; *p* = 0.4; 130 patients: MD −0.73; 95% CI −2.01, 0.54; *p* = 0.26, respectively). This was also reflected in the reported patient’s perception of satisfaction of treatment (147 patients: OR 3.74; 95% CI 0.93, 15.09; *p* = 0.06).

No serious adverse events were recorded by the included studies. More specifically, data concerning adverse events were available in four of the studies. As presented in Table 3, the most common symptoms included vaginal bleeding or spotting, discharge, pain/irritation or discomfort and lower urinary tract symptoms, and these were detected in both groups.

### 3.6. Sensitivity Analysis

The mean differences in VHI became significant in favor of laser CO_2_ after exclusion of the study by Page et al. (160 patients: MD −1.85; 95% CI −2.10, −1.60, *p* < 0.00001), and the same was observed in the proportion of satisfied patients (89 patients: MD 8.35; 95% CI 1.21, 57.91; *p* = 0.03) [18]. Moreover, as previously noted, the mean difference in FSFI scores was also significant upon exclusion of the study by Mension et al. (134 patients: MD 6.29; 95% CI 0.2, 12.37; *p* = 0.04) [17].

## 4. Discussion

The findings of the present meta-analysis revealed comparable outcomes in terms of both subjective (FSFI, patient satisfaction and VAS for dyspareunia) and objective parameters (VHI and pH) among patients who received laser CO_2_ treatment and sham control. The outcome that presented superiority in the laser CO_2_ group was the estimation of vaginal assessment scale score for the GSM symptoms. As already mentioned, this score aims to interpret the severity of some of the most bothersome GSM symptoms, as they are scaled by the patients. As a result, the significant improvement of this score in favor of laser CO_2_ over sham control should be taken into account when evaluating the efficacy of laser CO_2_ in GSM patients.

Despite the fact that changes in the FSFI scores did not differ among the two groups, the mean difference in FSFI score was significantly elevated in the laser CO_2_ group when the study by Mension et al. was excluded following sensitivity analysis. This could potentially be attributed to the design and inclusion criteria of the study. More specifically, in their study, patients in both groups were counseled on the use of non-hormonal lubrication and vaginal vibrator before the initiation of the study protocol, an intervention that could have influenced the laser efficacy [17,28]. Crucially, the study focused solely on breast cancer survivors under aromatase inhibitor therapy. Therefore, these women might suffer from more severe vaginal atrophy due to significantly decreased serum estradiol levels [29]. In addition to this, we should also take into account the significant impact of the surgical and overall breast cancer therapy on sexual function that is mainly due to the negative impression of the body image of breast cancer survivors and definitely makes them a population that is challenging to handle [30]. The effect of vaginal laser for the management of breast cancer survivors with GSM has been proved by a recent meta-analysis including a total of 10 observational studies that evaluated the improvement before and after treatment [31]. In particular, VHI, dyspareunia, and vaginal dryness were all found to be improved after treatment with laser CO_2_. However, the aforementioned outcomes are based on observational studies without control groups, and the study by Mension et al. is the only currently available one comparing the application of laser CO_2_ with a sham control [17]. This highlights the importance of further studies to elucidate whether the use of laser CO_2_ could improve GSM symptoms in this special population [29,32].

Moreover, the outcomes by Page et al. should also be interpreted with caution. The authors found no differences among all parameters between patients who received laser CO_2_ treatment and controls [18]. The exclusion of this study from the meta-analysis resulted in significant outcomes in both VHI and patient satisfaction. A letter to the editor by Salvatore et al. indicated that the abovementioned study had several technical, statistical, and patient selection issues that require caution in the interpretation of the study outcomes [33]. In summary, the use of SmartStack 2 in all sessions, the use of energy even in the sham control group, the definition and analysis based on the most bothersome symptom, and the short follow-up of 3 months after the last treatment are considered the most critical.

The technical aspects of laser CO_2_ application also remain debated. The literature shows considerable variation in the number of laser sessions and the power utilized. In the present study, the number of laser sessions varied between three and five (as shown in Table 1), and the power applied was either 30 or 40 watts. The prospective study by Athanasiou et al. showed that the addition of a fourth or fifth session may further enhance the therapeutic effect by increasing the proportion of symptom-free patients [34]. Patients with more pronounced sexual dysfunction seem to benefit by adding one or two extra sessions beyond the standard three sessions, despite the fact that no data from RCTs are available on the optimal number of sessions that could benefit each patient. The same study group observed no differences in the improvement of GSM symptoms after three monthly sessions, regardless of whether 30 or 40 watts of power was used for CO_2_ laser treatment [35].

Multiple systematic reviews and meta-analyses have been published with an aim to evaluate the potential effect of vaginal CO_2_ laser in patients with GSM. Among them, a systematic review and meta-analysis of RCTs by Khamis et al. compared the application of CO_2_ to sham control [8]. Three studies with 164 patients were included, and the outcomes revealed the superiority of laser CO_2_ in terms of VAS, FSFI, Urogenital Distress Inventory-6 (UDI-6), and patient satisfaction. In particular, VAS and UDI-6 were significantly reduced (*p* = 0.0004 and *p* = 0.03, respectively), FSFI score was improved (*p* < 0.00001), and a significantly increased number of patients in the laser group (*p* = 0.0004) were satisfied after the procedure. The aforementioned outcomes are not in accordance with the outcomes of the present study. This can be attributed to the number of included studies, the number of women included, as well as the analyzed parameters. Indeed, in the current updated meta-analysis, both the number of studies and of patients have doubled, while an analysis of VHI and vaginal pH was also conducted. Another meta-analysis by Filippini et al. included studies that compared outcomes of patients with GSM before and after the applications of a CO_2_ laser without the inclusion of a sham control [32]. The authors found a significant improvement in GSM symptoms (dyspareunia, dryness, itching, burning, and dysuria) and FSFI scores before and after laser therapy [36]. However, when compared with treatment with vaginal estrogens, laser CO_2_ was inferior in improving VAS scores, FSFI, VHI, and vaginal maturation index (VMI), as proved by the meta-analysis of six RCTs by Jang et al. [7].

### Strengths and Limitations

We aimed to eliminate data losses by removing data restrictions while three authors independently searched the literature. To our knowledge, this is the latest study to present outcomes from the currently available RCTs on the use of a vaginal CO_2_ laser compared with sham control in patients with GSM.

However, a number of limitations need to be addressed. First of all, the number of existing studies and, as a result, of recruited patients is still limited. Consequently, the limited body of evidence may potentially affect the reliability and generalizability of the findings regarding the effect of the laser treatment, precluding and thus reaching robust conclusions.

Furthermore, despite their methodological quality, the recruited studies presented differences in design, methodology, and included populations. More specifically, as shown in Table 1, significant heterogeneity was detected in a number of laser sessions as well as in the intervals among them and the technical aspects of laser (such as power and stack mode) that were adopted by each study. Finally, the inclusion criteria and the pre-treatment vaginal preparation also seem to be variable among the studies. Further well-designed randomized trials are warranted so as to clarify the aforementioned limitations and to allow us to draw safe conclusions.

## 5. Conclusions

The use of vaginal CO_2_ laser seems to be a safe and beneficial alternative for the management of symptoms of GSM. While the CO_2_ laser demonstrated similar outcomes to sham controls when comparing parameters such as the FSFI and VAS for dyspareunia, the sensitivity analysis revealed exceptions, where FSFI, VHI, and patient satisfaction yielded superior results favoring the laser treatment. Despite the promising outcomes, the significant heterogeneity among the studies concerning the number of laser sessions, technical aspects of laser application, and patient selection criteria underscores the need for further robust, well-designed randomized trials. Future study in the field should solidify the evidence base, investigate long-term outcomes, and validate the safety and efficacy of laser CO_2_ in GSM, while simultaneously providing clearer guidance for clinical practice.

## Figures and Tables

**Table 1:** Main characteristics of the included studies.

Year; Author	Country	Type of Study	Inclusion Criteria	Treatment Details	Outcomes Measured	Type of Laser
2023; Mension [17]	Spain	DB-RCT	Breast cancer survivors, age ≥ 30 yrs, aromatase inhibitors (for ≥6 months), menopause, signs or symptoms of GSM with dyspareunia, vaginal pH ≥ 5, willingness for sexual activity; no use of vaginal lubricants or moisturizers for 30 days; no vaginal hormonal treatment for 6 mo; no radiofrequency, laser treatment, hyaluronic, or lipofilling in the vagina for 2 years; no ospemifene treatment; no intraepithelial neoplasm of cervix, vagina, or vulva; no active genital tract infection; no prior treatment for genital cancer; no organ prolapse ≥ stage II; no positive test results for human papillomavirus	5 sessions, 4-week intervals	FSFI, dyspareunia, body image, quality of life, VHI, vaginal pH, VMI, VET, VEE, adverse effects, tolerance	Microablative CO_2_ laser system, SmartXide2 V^2^LR, MonaLisa Touch (DEKA Laser) with 40 W power, 1000 μs dwell time, 1000 μm dot spacing, SmartStack 2 on double pulse emission mode
2023; Page [18]	Belgium	DB-RCT	Moderate-to-severe GSM symptoms; MBS score ≥ 2; no acute or recurrent urogenital infections; no prolapse grade ≥ 3; no hormonal replacement therapy for the last 6 months; no vaginal estrogen, moisturizers, lubricants, homeopathic preparations, or physiotherapy for pelvic floor disorders for the last 3 months; no previous vaginal laser therapy	3 sessions, 4-week intervals	Relief of most bothersome symptom (dryness, itching, burning, dyspareunia, dysuria)—scale 0–3, VAS, patient satisfaction, FSFI, ICIQ-OAB, adverse events, VHI, vaginal pH, vaginal architecture	Fractional microablative CO_2_, SmartXide2 V2LR Monalisa Touch (DEKA, Florence, Italy) laser with 30 W power, 1000 ms dwell time, 1000 mm dot spacing, SmartStack 2.0
2021; Cruff [14]	USA	DB-RCT	Menopausal or post bilateral oophorectomy status; moderate-to-severe dyspareunia or vaginal dryness; vaginal health index (VHI)<15 and vaginal pH > 5; POP-Q stage < III; no pelvic reconstructive surgery 6 months before; no malignancy; no acute or recurrent genital tract infections; no serious diseases or chronic conditions; no estrogen use for 3mo prior; no use of moisturizers, lubricants, or homeopathic preparations in past 14 days; willingness to discontinue lubricants or estrogens	3 sessions, 6-week intervals	FSFI, DIVA, UDI-6, PGI-I, VAS for GSM symptoms, dyspareunia	Fractional microablative CO_2_ laser MonaLisa Touch Vagina: 30 W power, 1000 ms dwell time, 1000 mm dot spacing, SmartStack 1–3 Vulva: 26 W power, 800 ms, 800 mm dot spacing, SmartStack 1
2021; Li [15]	Australia	DB-RCT	Age ≥18 years; no previous vaginal energy-based treatment for GSM; amenorrhea ≥ 12 mo (naturally or iatrogenically); vaginal symptoms: dyspareunia, burning, itching, or dryness; ineffective previous treatment or contraindicated (personal lubricants, vaginal moisturizers, or estrogen); discontinuation of vaginal estrogen for 6 months before inclusion; no prolapse ≥ stage II; no active genital or urinary tract infections; no previous vaginal mesh surgery; no ongoing medical conditions	3 sessions, 4-week intervals (min 4 weeks; max 8 weeks)	Change in symptom severity, dyspareunia, dysuria, vaginal dryness, burning, itching (VAS), VSQ, VHI, VMI, vaginal biopsy	Laser CO_2_ (SmartXide V2LR, MonaLisa Touch, DEKA Laser) 40 W power, 1000 μs dwell time, 1000 μm dot spacing, SmartStack 2 on DP emission mode, delivering fluence of 5.37 J/cm^2^
2020; Ruanphoo [13]	Thailand	DB-RCT	Age ≥ 50 years; last menstruation at least 1 year ago; no hormonal therapy within the past 6 months; no vaginal moisturizer or lubricant for 30 days; no acute/recurrent urinary tract infection; no active genital infection; genital hiatus ≥ 2 cm; no prolapse stage ≥ 2	3 sessions, 4-week intervals	VAS for symptoms, vaginal health index, ICIQ-VS, adverse events, patient satisfaction	The laser settings were DEKA pulse mode, 40 W power, 1000 ms dwell time, 1000 mm dot spacing, SmartStack parameter 1–3
2021; Salvatore [16]	Italy, Greece	DB-RCT	Postmenopausal women with dryness and dyspareunia related to GSM; no vulvodynia; no vulvovaginitis; no vulvovaginal pathology; no prior treatment with energy-based devices; no use of non-hormonal/hormonal local therapies; no prolapse stage > 2	3 sessions, 4-week intervals	VAS, FSFI, UDI-6, changes in dryness, dyspareunia	Microablative fractional CO_2_ laser (SmartXide2 V2LR Monalisa Touch; DEKA, Florence, Italy) Vagina: 30 W power, 1000 ls dwell time, 1000 lm dot spacing, SmartStack 1–3; D-pulse mode; pulse energy, 43.2 mJ, 86.4 mJ, and 129.6 mJ at the 1st, 2nd, and 3rd sessions, respectively; Introitus and labia minora: 24 W power, 400 ls dwell time, 1000 lm spacing, SmartStack parameter 1; D-pulse mode; fluence, 2.36 J/cm^2^; pulse energy, 23.2 mJ
2020; Quick [12]	USA	SB-RCT	History of cervical, endometrial, vaginal, vulvar or ovarian cancer with dyspareunia and/or vaginal dryness, unable to be sexually active due to pain, completed all cancer-related treatment prior 6 months, no recurrent or metastatic cancer, no prolapse stage ≥ 2, no prior reconstructive pelvic surgery with mesh, no hormone therapy 6 weeks before treatment	3 sessions, 4-week intervals	VAS, vulvar assessment scales, FSFI, UDI-6, patient satisfaction, adverse events	Fractional microablative CO_2_ (Monalisa Touch, DEKA, Florence, Italy) Vagina: 30 W power, 1000 μs dwell time, 1000 mm dot spacing, SmartStack 1 and 3 Vestibule: 26 W power, 800 μs dwell time, 800 μm dot spacing, SmartStack 1

DB-RCT: double-blind randomized controlled trial; SB-RCT: single-blind randomized controlled trial; GSM: genitourinary syndrome of menopause; FSFI: female sexual function index score, VHI: vaginal health index, VMI: vaginal maturation index, VET: vaginal epithelial thickness, VEE: vaginal epithelium elasticity, VSQ: vulvovaginal symptom questionnaire.

**Table 2:** Main characteristics of the included patients.

Year; Author	Patient No	Age (Years)	Parity	Sexually Active (Initially)	Years of Menopause	Iatrogenic-Induced Menopause	Lubrication Use/MHT	Follow-Up (Months)
2023; Mension [17]	CLT (CO_2_) vs. SLT (sham) 35 vs. 37	51.3 ± 7.8 vs. 53.7 ± 8.8	N/A	25 vs. 27	N/A	26 vs. 20	N/A	6 months
2023; Page [18]	29 vs. 29	57.4 ± 7.07 vs. 56.2 ± 6.3	N/A	N/A	6.85 ± 5.41 vs. 7.3 ± 5.22	9 vs. 9	5 vs. 5	3 months
2021; Cruff [14]	12 vs. 16	61 (54-66) vs. 59 (56-65) ^a^	2 (2–3) vs. 2 (2–3)	12 vs. 13	14 (5–24) vs. 10 (4–15) ^a^	N/A	5 vs. 5 (estrogen)	6 months
2021; Li [15]	43 vs. 42	55 ± 7 vs. 58 ± 8	4 vs. 6 nulliparous	23 vs. 21	8 (4–14) vs. 6 (3–9) (median IQR)	20 vs. 21	N/A	12 months
2020; Ruanphoo [13]	44 vs. 44	61.73 ± 8.01 vs. 59.84 ± 7.49	2.11 ± 1.51 vs. 2.20 ± 1.53	10 vs. 24	N/A	N/A	N/A 10 vs. 8	3 months
2021; Salvatore [16]	28 vs. 30	57 ± 6.9 vs. 58.4 ± 6	N/A	N/A	N/A	N/A	N/A	4 months
2020; Quick [12]	10 vs. 8	56 ± 11.17 vs. 56.8 ± 5.95	N/A	N/A	N/A	N/A	1 vs. 1 N/A	4 months

Continuous data are reported in mean ± SD except in ^a^ median (95% CI); MHT: menopausal hormone therapy; N/A: not available.

**Table 3:** Main outcomes of the included studies.

Year; Author	Patient No	Vaginal Assessment Scale for GSM Symptoms *	UDI-6 *	FSFI *	VHI *	VAS Dyspareunia (Scale 0–10) *	Satisfaction (Patient No) **	Vaginal pH *	Adverse Events
2023; Mension [17]	35 vs. 37	N/A	N/A	5.2 ± 1.5 ^a^ vs. 7.9 ± 1.2 ^a^	3.3 ± 4.1 ^a^ vs. 5 ± 4.5 ^a^	−4.3 ± 3.4 ^a^ vs. −4.5 ± 2.3 ^a^	N/A	−0.6 ± 0.9 ^a^ vs. −0.8 ± 1.2 ^a^	N/A
2023; Page [18]	29 vs. 29	−0.61 ± 0.84 ^a^ vs. −0.364 ± 0.73 ^a^	N/A	3.51 ± 6.22 ^a^ vs. 3.14 ± 7.71 ^a^	2.9 ± 4.21 ^a^ vs. 1.24 ± 4.23 ^a^	−2.31 ± 4.51 ^a^ vs. −2.17 ± 3.45 ^a^	12/29 vs. 10/29	0.02 ± 0.64 ^a^ vs. 0.12 ± 0.44 ^a^	No serious, minor vaginal bleeding, spotting or discharge
2021; Cruff [14]	12 vs. 16	N/A	−18.8 (−37.5 to 8.3) ^b^ vs. −8.3 (−16.7 to 8.3) ^b^	6.4 (−2.1 to 17.7) ^b^ vs. 6.6 (2.8 to 12.3) ^b^	3 (0 to 6) ^b^ vs. 5 (0 to 7) ^b^	N/A	N/A	N/A	No
2021; Li [15]	43 vs. 42	N/A	N/A	N/A N/A	0.9 (−2.2 to 4) ^c^ vs. 1.3 (−1.4 to 4) ^c^	−28.8 (−67.7 to 10) ^c^ vs. −4 (−35.3 to 27.4) ^c^ (scale 0−100)	N/A	N/A	16 vs. 17 (vaginal pain/discomfort, spotting, lower urinary tract symptoms, lower or upper urinary tract infection, vaginal discharge)
2020; Ruanphoo [13]	44 vs. 44	−0.44 ± 0.66 ^a^ vs. 0.04 ± 0.63 ^a^	N/A	N/A	−3.27 ± 0.78 ^a^ vs. −1.42 ± −0.36 ^a^	N/A	31/39 vs. 17/38	N/A	Vaginal bleeding 0 vs. 1 Vaginal discharge 3 vs. 1 Vaginitis 1 vs. 0 Pain after procedure 3 vs. 4
2021; Salvatore [16]	28 vs. 30	N/A	−8 ± 15.3 ^a^ vs. −2.6 ± 9.6 ^a^	12.3 ± 8.9 ^a^ vs. 2.4 ± 4.9 ^a^	N/A	−6 ± 2.6 ^a^ vs. −1.1 ± 1.8 ^a^	N/A	N/A	Mild vulva irritation 28 (laser group)
2020; Quick [12]	10 vs. 8	−3 ± 1.7 ^a^ vs. −2 ± 3.5 ^a^	−25 ± 28.3 ^a^ vs. −4.18 ± 13.3 ^a^	7.025 ± 5.51 ^a^ vs. −1.68 ± 3.4 ^a^	N/A	N/A	6/6 vs. 1/6	N/A	N/A

^a^ Mean ± SD, ^b^ median (95% CI), ^c^ mean 95% CI, N/A: not available, GSM: genitourinary syndrome of menopause, FSFI: female sexual function index score. * The marked scores are presented as difference between baseline and follow-up for each group. The negative outcome indicates the respective decline in the parameter with this score. ** Satisfaction refers to the number of patients that stated they were “satisfied” or “very satisfied” with the procedure.

## Data Availability

No new data were created or analyzed in this study. Data sharing is not applicable to this article.
